# Clinicopathological features of hepatoid adenocarcinoma of the stomach: A multicenter retrospective study

**DOI:** 10.1002/cnr2.2101

**Published:** 2024-06-03

**Authors:** Senichiro Yanagawa, Kazuaki Tanabe, Mikihiro Kano, Ryuichi Hotta, Yoshihiro Saeki, Nobuaki Fujikuni, Hideki Ohdan, Hiroshima Surgical Study Group of Clinical Oncology (HiSCO), Noriaki Tokumoto, Toshihiro Misumi, Hirofumi Tazawa, Takahisa Suzuki, Kazuhiro Toyota, Yoichi Sugiyama, Toshikatsu Fukuda, Masahiro Nishihara, Masahiro Ikeda, Yasuhiro Imaoka, Masayuki Shishida, Emi Chikuie, Yuji Yamamoto

**Affiliations:** ^1^ Department of Surgery JA Onomichi General Hospital Hiroshima Japan; ^2^ Department of Perioperative and Critical Care Management Graduate School of Biomedical and Health Sciences, Hiroshima University Hiroshima Japan; ^3^ Department of Surgery Hiroshima City North Medical Center Asa Citizens Hospital Hiroshima Japan; ^4^ Department of Surgery National Hospital Organization Higashihiroshima Medical Center Higashihiroshima Japan; ^5^ Department of Gastroenterological and Transplant Surgery Hiroshima University Hiroshima Japan; ^6^ Department of Digestive Surgery Hiroshima Prefectural Hospital Hiroshima Japan

**Keywords:** chemotherapy, gastrectomy, hepatectomy, hepatoid adenocarcinoma of the stomach, liver metastasis

## Abstract

**Background:**

Hepatoid adenocarcinoma of the stomach (HAS) is a rare and aggressive subtype of gastric cancer (GC), accounting for less than 1% of all cases. It is characterized by frequent liver metastasis recurrence and a poorer prognosis than conventional GC. However, established treatment guidelines for HAS are currently not available.In this report, we present the results of a clinicopathological study of 19 patients diagnosed with HAS, including seven patients with liver metastasis, conducted by the Hiroshima Surgical Study Group of Clinical Oncology (HiSCO) between 2016 and 2018.

**Aims:**

The aim of the study was to retrospectively observe the outcomes of HAS with gastrectomy and hepatectomy for liver metastasis and determine relevant prognostic factor. We also examined the criteria and outcomes of hepatectomy for liver metastasis and aimed to suggest the optimal treatment for HAS, including chemotherapy.

**Methods and Results:**

A total of 2147 patients underwent gastrectomy for GC at HiSCO‐affiliated institutions during the study period; 19 patients, all male with a mean age of 70.9 years, were diagnosed with HAS by hematoxylin‐eosin and immunohistochemical staining. Patients underwent gastrectomy at varying pathological stages: six at Stage I, three at Stage II, seven at Stage III, and three at Stage IV. Ten patients received postoperative chemotherapy and the 5‐year survival rate was 67.7% after gastrectomy. Among the seven patients with pre or postoperative liver metastasis, five patients underwent hepatectomy. Although one patient had recurrence, the 3‐year survival rate was 100% after hepatectomy.

**Conclusion:**

Contrary to previous reports suggesting a 3‐year survival rate of approximmately 30% for HAS, our findings indicate that the prognosis for HAS may not be as poor as reported previously. This study contributes valuable insights into the management and potential treatment strategies for HAS.

**Abbreviations**
AFP: alpha‐fetoprotein
CA19‐9: carbohydrate antigen 19‐9
CapeOX: capecitabine with oxaliplatin
CEA: carcinoembryonic antigen
CT: computed tomography
CTCAE: common terminology criteria for adverse events
DG: distal gastrectomy
DS: S1 with docetaxel
GC: gastric cancer
HAC: hepatoid adenocarcinoma
HAS: hepatoid adenocarcinoma of the stomach
H.E: hematoxylin and eosin
HiSCO: Hiroshima Surgical Study Group of Clinical Oncology
ICG‐R15: indocyanine green retention test at 15 min
MRI: magnetic resonance imaging
PET‐CT: positron emission tomography
PPG: pylorus‐preserving gastrectomy
RFA: radiofrequency ablation
SOX: S1 with oxaliplatin
TG: total gastrectomy
TRG: tumor regression grade

## 1 INTRODUCTION

Hepatoid adenocarcinoma (HAC) is a rare histologic subtype of hepatocellular carcinoma that has been identified in various organs, including the stomach, ovaries, renal pelvis, papilla of Vater, lungs, and pancreas.^1^, ^2^ HAC is defined as adenocarcinoma of extrahepatic origin with morphological features of liver cell differentiation, composed of large polygonal eosinophilic hepatocytes. When HAC occurs in the stomach, it is referred to as hepatoid adenocarcinoma of the stomach (HAS) according to the 4th edition of the World Health Organization classification of the tumors of the digestive system.^3^ HAS comprises a very small proportion of malignant gastric tumors and accounts for only 0.3% of all gastric adenocarcinoma cases.^4^, ^5^

HAS is tumor tissue consisting of large eosinophilic cells exhibiting trabeculae separated by sinusoidal vascular channels in hematoxylin and eosin (H.E) staining. Typically, it presents with high levels of alpha‐fetoprotein (AFP)^6^, ^7^ and can be confirmed by several immunohistochemical markers, such as AFP, Glypican‐3, Sal‐like protein 4, and Hep‐Par 1. This subtype of gastric cancer (GC) frequently tends to present with liver metastases and carries a poor prognosis. The literature reports that patients with HAS have an average survival period of 10–18 months, with 1‐, 3‐, and 5‐year survival rates ranging from 30% to 37.5%, 7% to 13%, and 8.3% to 9%, respectively.^8^

Currently, the mechanisms underlying the development and progression of HAS remain unknown, and no specific guidelines for HAS treatment exist. The surgical procedures and chemotherapy used for treating conventional GCs have been applied to HAS. However, HAS has a higher rate of liver metastasis (35%–76%) than peritoneal dissemination recurrence,^9^, ^10^ and the establishment of an effective treatment modality for liver metastasis would improve the poor prognosis.

In this report, we present the results of a clinicopathological study of 19 patients diagnosed with HAS, including seven patients with liver metastasis, conducted by the Hiroshima Surgical Study Group of Clinical Oncology (HiSCO). This study aimed to retrospectively observe the outcomes of HAS with gastrectomy and hepatectomy for liver metastasis and determine relevant prognostic factor. We also examined the criteria and outcomes of hepatectomy for liver metastasis and aimed to suggest the optimal treatment for HAS including chemotherapy.

## 2 MATERIALS AND METHODS

### 2.1 Patients and diagnosis of HAS

**FIGURE 1:**
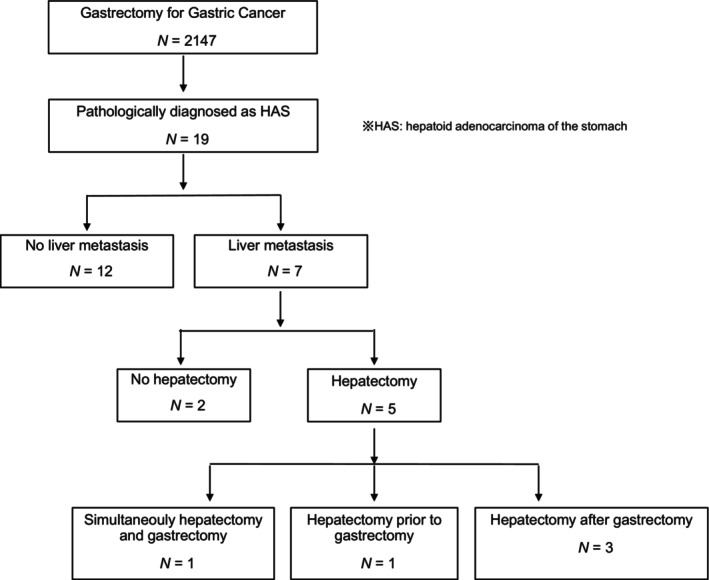
Flow chart of participant inclusion in the present study.

Between January 2016 and December 2018, a total of 2147 patients underwent gastrectomy for GC at 14 HiSCO institutions. Among these patients, 19 (0.88%) showed the following features: large, irregular cancer cells with abundant cytoplasm, that exhibited pale eosin staining and a medullary or cable structure, formed by fibrous tissues and a rich blood supply as observed with H.E staining and positive immunohistochemical staining for AFP. These were then diagnosed as HAS by pathologists at each institution (Figure 1) according to the literature.^3^, ^11^ Clinical and pathological data for all 19 patients were extracted from medical records.

### 2.2 Diagnosis of liver metastasis and criteria for hepatectomy

Among the 19 patients, seven had liver metastases; five underwent hepatectomy, while two did not. Two of the five patients had liver metastases at the time of GC diagnosis: one underwent simultaneous gastrectomy and hepatectomy, and the other underwent hepatectomy followed by gastrectomy. Three of the five patients experienced liver metastasis recurrence after gastrectomy and subsequently underwent hepatectomy (Figure 1). The liver metastasis was diagnosed by images of computed tomography (CT) and magnetic resonance imaging (MRI) or positron emission tomography CT (PET‐CT). Liver metastases appeared as low‐density areas in the portal vein contrast phase in CT and the hepatocyte contrast phase in MRI, and they appeared distinct from primary liver cancer.

The criteria for hepatectomy were as follows: preserved hepatic function (Child‐Pugh A/B), no HAS metastasis to organs other than the liver, three or fewer tumor units, and portal vein invasion up to the first bifurcation, accordance with the Japanese guidelines for liver cancer treatment.^12^ Age and tumor diameter were not included in the criteria.

### 2.3 Assessment for adverse events of chemotherapy

Adverse events to chemotherapy administration were evaluated using the Common Terminology Criteria for Adverse Events (CTCAE) v4.0.

### 2.4 Statistical analysis

Survival rates were estimated by Kaplan–Meier survival curves, employing EZR (Saitama Medical Centre, Jichi Medical University, Saitama, Japan), a graphical user interface for R (The R Foundation for Statistical Computing, Vienna, Austria). Specifically, EZR is a modified version of the R commander designed to incorporate statistical functions frequently used in biostatistics.^13^ Statistical significance was defined at the level of *p* < .05.

## 3 RESULTS

### 3.1 Patient characteristics

A summary of the 19 cases diagnosed as HAS is presented in Table 1. The mean age of the patients was 70.8 years, and all patients were male. Prior to gastrectomy, the mean serum carcinoembryonic antigen (CEA) level was 7.1 ± 3.8 ng/mL (range 0.8–74.9: reference range: 0–5.0 ng/mL), the carbohydrate antigen 19‐9 (CA 19‐9) level was 8.6 ± 1.5 U/mL (range 2–25.6: reference range: 0–37.0 U/mL), and serum AFP level was 159.3 ± 28.9 ng/mL (range 109–294.5: reference range: 0.89–8.78 ng/mL). The results of preoperative histopathological biopsy revealed the intestinal type in eight cases, diffuse type in eight cases, and indeterminate type in three cases.

**TABLE 1:** Baseline characteristics of 19 cases of hepatoid adenocarcinoma of stomach.

Parameter		*N* = 19
Age		70.9 ± 1.8
Male/female		19/0
Serum CEA (ng/mL)		7.1 ± 3.8 (0.8–74.9)
Serum CA19‐9 (U/mL)		8.6 ± 1.5 (2–25.6)
Serum AFP (ng/mL)		159.3 ± 28.9 (109–294.5)
Surgical procedure	DG/L‐PPG/TG	13/2/4
Tumor location	Fundus/corpus/antrum and pylorus	3/8/8
Type	0/I/II/III/IV/V	9/2/5/2/0/1
Diameter (mm)		52.1 ± 7.9
Histopathology	Intestinal type	8
	Diffuse type	8
	Indeterminate	3
pT	1	8
	2	3
	3	6
	4	2
pN	0	6
	1,2,3	13
Ly	0	4
	1a,1b,1c	15
V	0	5
	1a,1b,1c	14
pStage	I	6
	II	3
	III	7
	IV	3
Residual tumor	0/1	17/2
Recurrence	+/−	7/12
Outcome	Alive/dead	14/5

*Note*: Data are presented as mean ± standard deviation.

Abbreviations: AFP, alpha‐fetoprotein; CA19‐9: carbohydrate antigen 19‐9; CEA, carcinoembryonic antigen; DG, distal gastrectomy; PPG, pylorus‐preserving gastrectomy; TG, total gastrectomy.

Four patients underwent total gastrectomy (TG), 13 underwent distal gastrectomy (DG), and two underwent pylorus‐preserving gastrectomy (PPG). Liver metastases were detected before gastrectomy in two cases. Pathologically, the rate of lymph node metastasis was 68.4% (13/19), lymphatic invasion 78.9% (15/19), and venous invasion 73.7% (14/19). The final pathological diagnoses were pStage I in six cases, pStage II in three cases, pStage III in seven cases, and pStage IV in three cases, according to the Union for International Cancer Control tumor node metastasis classification.^14^ R0 surgery was performed in 17 cases (89.5%).

During the observation period, recurrence was observed in seven patients, and three patients died.

### 3.2 Cases with chemotherapy

Adjuvant chemotherapy for pStage II or III was administered in seven cases and chemotherapy for pStage IV was administered in three cases (Table 2). In pStage II and III patients, two patients received capecitabine with oxaliplatin (CapeOX) according to the CLASSIC trial protocol as adjuvant chemotherapy.^15^ One completed the course and one stopped oxaliplatin after the third course due to the adverse events of peripheral neuropathy (Grade 3 by CTCAE) and only capecitabine was continued. Four patients received S1 according to the ACTS‐GC protocol,^16^ of whom treatment was completed in three patients, but one patient stopped after the second course due to the adverse events of skin disorder (Grade 2 by CTCAE). One patient received S1 with docetaxel (DS) according to the JACCRO GC‐07 protocol^17^ and the treatment was completed. In pStage IV, all three patients received S1, two patients were shifted to second‐line treatment because of recurrence and died due to disease progression. One patient is still on S1 without recurrence.

**TABLE 2:** Regimen and pStage of 10 cases.

pStage	Regimen	*N*
II and III	CapeOX	2
	S1	4
	DS	1
IV	S1	3

Abbreviations: CapeOX, capecitabine with oxaliplatin; DS, S1 with docetaxel.

### 3.3 Overall survival rate after gastrectomy and recurrence rate

**FIGURE 2:**
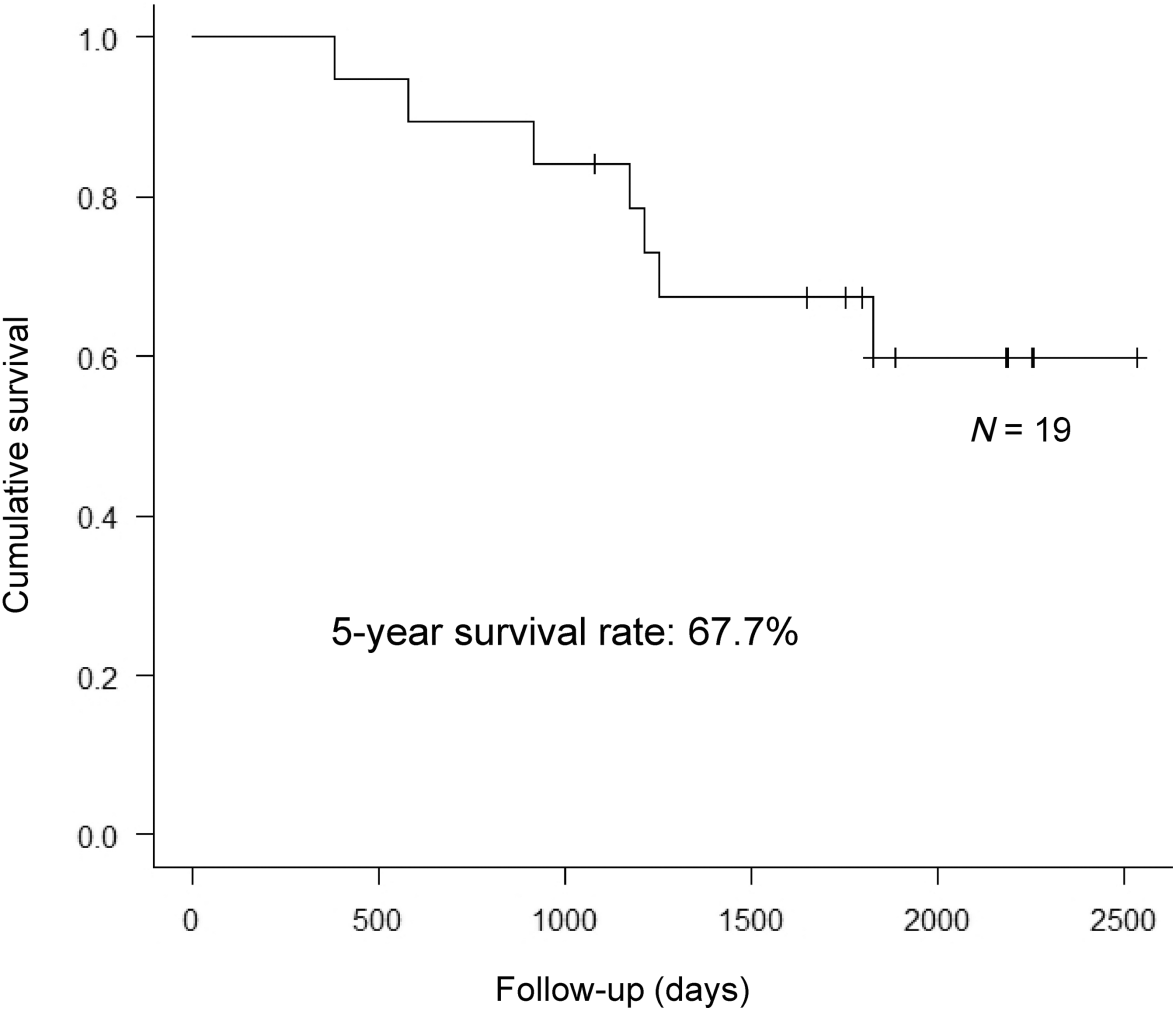
Overall survival after gastrectomy: 1‐year survival rate 100%, 3‐year survival rate 78.9%, and 5‐year survival rate 67.7%.

**FIGURE 3:**
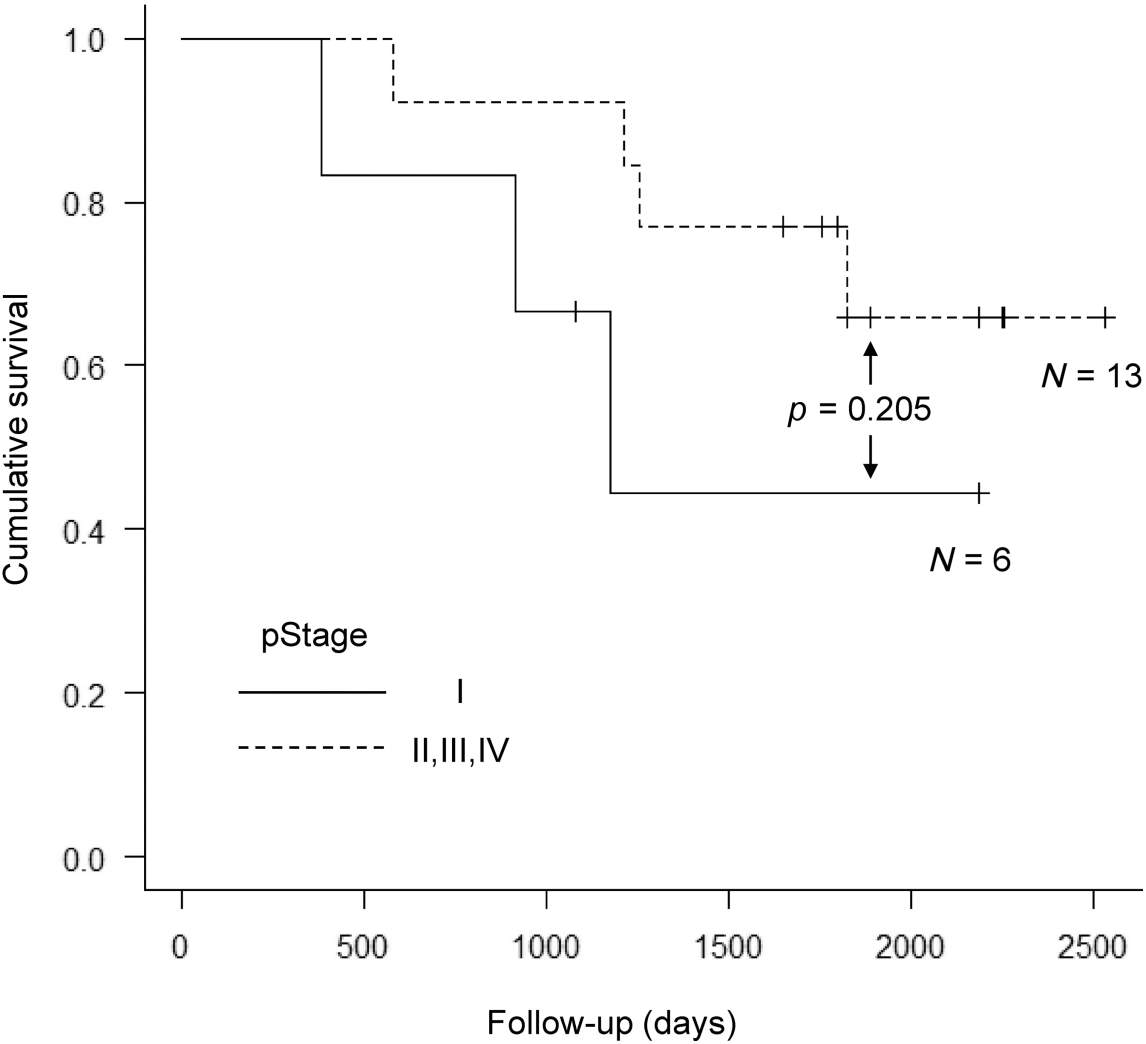
Comparison of survival rate between pStage I and II, III, and IV. There was no statistically significant difference in the 5‐year survival rate between the two groups (*p* = .205).

The overall survival rate after gastrectomy was 100% at 1 year, 78.9% at 3 years, and 67.7% at 5 years (Figure 2). All patients were followed up at the institution where the surgery was performed. The median follow‐up duration was 4.9 (range 1.1–6.9) years for all patients. Among the six patients in pStage I, two exhibited recurrence (33.3%), and among the 13 patients in pStage II, III, IV, five exhibited recurrence (38.5%). There was no statistical difference in recurrence rate between the two groups (*p* = 1). The 5‐year survival rate of patients in pStage I was 44.4% and pStage II, III, IV was 76.9% (*p* = .205) (Figure 3). Two patients with pStage I died during the observation period. The one was pathologically diagnosed with the Intestinal type, T1b, N1, Ly1a, V1c and died from jaundice and liver failure due to multiple liver metastases 1 year after gastrectomy. The other patient was died of respiratory failure due to exacerbation of emphysema without recurrence of HAS.

### 3.4 Cases and survival rate with hepatectomy and efficacy of chemotherapy for liver metastasis

Of the 19 cases, liver metastasis was detected in seven cases on CT, MRI, or PET‐CT, of which hepatectomy was performed in five cases and two cases were deemed unresectable (Figure 1).

In pStage I, two patients with liver metastasis were detected, 1 patient could not be treated as resectable and one patient underwent CapeOX treatment followed by hepatectomy. In pStage II, two patients with liver metastasis were detected, of which one was not resectable, and one underwent hepatectomy after CapeOX treatment. In pStage III, one patient with liver metastasis was detected, and the patient underwent hepatectomy after CapeOX treatment. In pStage IV, two patients with liver metastasis at the time of diagnosis of GC underwent hepatectomy after chemotherapy (Table 3).

**TABLE 3:** Characteristics of liver metastasis cases with hepatectomy.

No	Number of metastasis (site)	Total tumor diameter (pre and post chemotherapy)	Regimen before hepatectomy	Child‐Pugh	ICG‐R15 (%)	Surgical procedure	TRG according to Becker	Recurrence post‐hepatectomy
1	2 (S5/6,7)	55 → 58 mm	CapeOX	A	6.4	Posterior segmentectomy	2	+
2	1 (S6)	49 → 22 mm	CapeOX	A	9	Posterior segmentectomy	2	−
3	1 (S6)	23 → 10 mm	CapeOX	A	9.1	Partial hepatectomy	1a	−
4	2 (S7)	141 → 65 mm	CapeOX	A	18.9	Subsegmentectomy	1a	−
5	2 (S5,6)	100 mm^a^	S1	A	14.2	Right hepatic lobectomy	1a	−

^a^ Prior hepatectomy for liver metastasis.

Abbreviations: CapeOX, capecitabine with oxaliplatin; ICG‐R15, indocyanine green retention test at 15 min; TRG, tumor regression grade.

**FIGURE 4:**
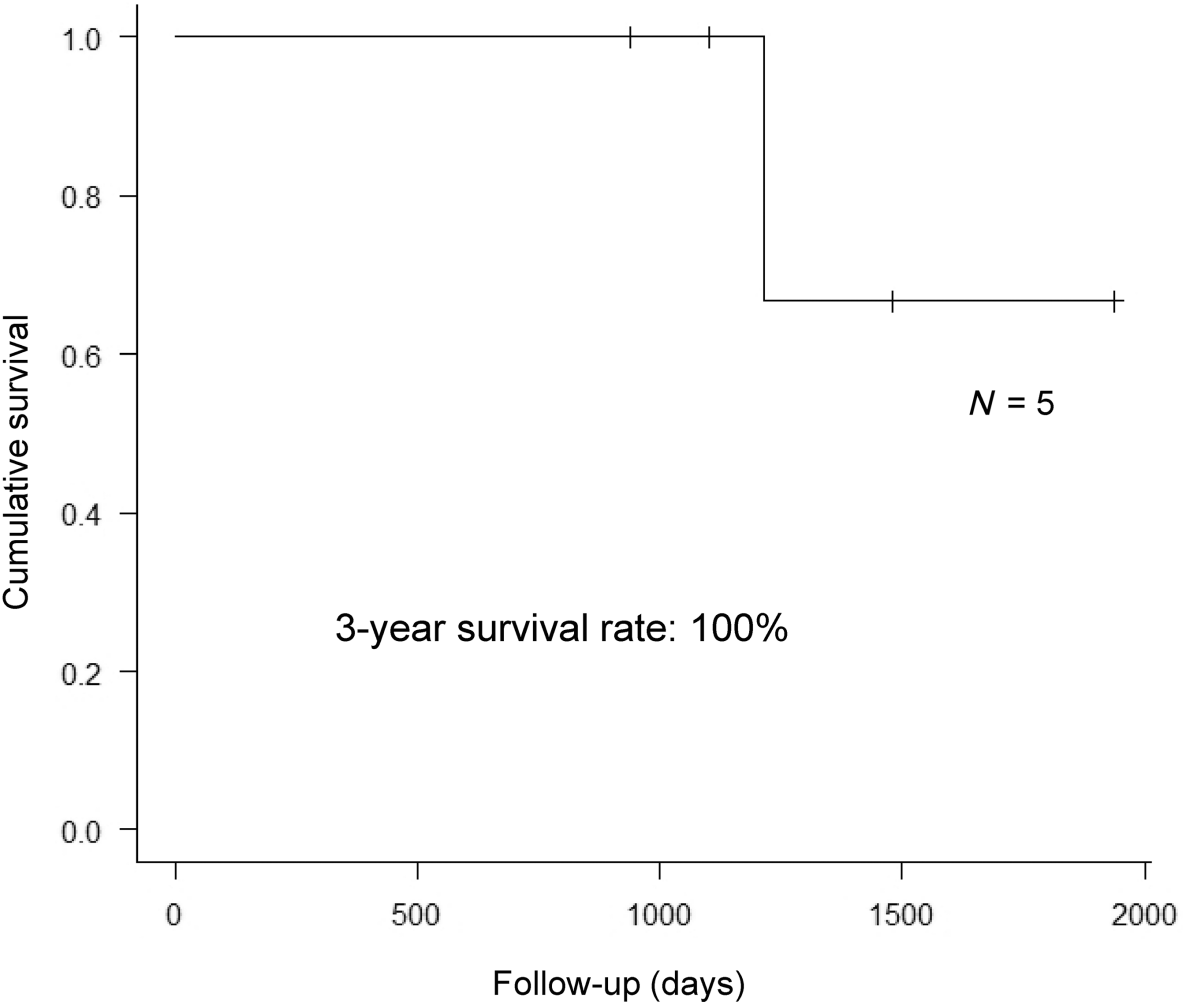
Overall survival after hepatectomy: 3‐year survival rate of five cases was 100%.

According to Becker's criteria, tumor regression grade (TRG) is determined on resected specimens of liver metastases and GC by pathologists at each institution.^18^, ^19^ In all cases, chemotherapy resulted in a pathologically effective response. The 3‐year survival rate for the five cases with hepatectomy was 100% (Figure 4).

The median follow‐up duration was 3.5 (range 2.6–5.3) years for all patients.

## 4 DISCUSSION

In 1985, Ishikura et al. reported the first case of HAS; the following year, they documented seven similar cases, highlighting that this tumor type primarily occurred in elderly individuals, often in the antrum, exhibited distinct hepatoid differentiation, frequently had liver metastasis, and was associated with a poor prognosis.^20^ Immunohistochemically, AFP is often distributed in hepatoid regions, and more is expressed by more than 90% patients, whereas it is weakly or focally positive in adenocarcinomatous areas.^5^, ^11^ AFP and hepatocyte antigens can be utilized to differentiate HAS from hepatocellular and common adenocarcinoma of the stomach.

HAS is a unique subtype of GC that frequently metastasizes to the liver. To the best of our knowledge, since 2010, according to original articles on HAS,^8^, ^9^, ^21^, ^22^, ^23^, ^24^, ^25^, ^26^, ^27^ reported cases have been from single centers (Table 4). The rate of preoperative liver metastasis of GC ranged from 2.7% to 17.6%, and postoperative liver metastasis of GC from 21.1% to 75.6%. The 5‐year survival rate ranged from 9% to 41.1% and has been reported as a poor prognosis carcinoma.^8^, ^9^, ^21^, ^22^, ^23^, ^24^, ^25^, ^26^ However, Zhou et al. reported a relatively favorable prognosis, with 1‐ and 3‐year survival rates of 87.9% and 82.6% after gastrectomy, respectively.^27^ Our study results showed that the 1‐, 3‐, and 5‐year survival rates post‐gastrectomy were 100%, 78.9%, and 67.7%, respectively, which is considered a relatively good prognosis, as reported by Zhou et al.

**TABLE 4:** Original article summaries of hepatic adenocarcinoma of the stomach since 2010.

Study year	Study scale	Number of cases	Liver metastasis before gastrectomy (%)	Liver metastasis after gastrectomy (%)	1‐year survival rate (%)	3‐year survival rate (%)	5‐year survival rate (%)
Zhang^21^ 2011	Single center	20	20	30	‐	17.2	‐
Liu^22^ 2012	Single center	45	‐	75.6	30	13	9
Yang^23^ 2014	Single center	31	17.6	41.9	‐	22.6	‐
Lin^24^ 2015	Single center	10	‐	40	‐	‐	20
Xie^9^ 2015	Single center	19	15.8	36.8	‐	30	30
Xiao^25^ 2015	Single center	9	0	21.1	‐	‐	‐
Wang^26^ 2019	Single center	42	‐	32.4	‐	‐	41.1
Zhang^8^ 2020	Single center	13	0	‐	70	30	‐
Zhou^27^ 2020	Single center	75	2.7	‐	87.9	82.6	‐
Present study	Multi center	19	5.2	15.8	100	78.9	67.7

Various drugs, such as platinum, antimetabolites, and molecular‐targeted agents, have been used for chemotherapy in prior reports, and transcatheter arterial chemoembolization or radiofrequency ablation (RFA) have been used for liver metastases, with no consensus on drug therapy.^5^, ^28^ Even when limited to adjuvant chemotherapy, several agents are employed, including S1 with oxaliplatin (SOX), 5‐fluorouracil/l‐leucovorin/oxaliplatin, S1, oxaliplatin/fluorouracil, cisplatin/cetuximab, and docetaxel.^9^, ^23^, ^26^, ^28^ However, standard neoadjuvant or adjuvant chemotherapy for HAS recommended by randomized controlled trials has not yet been established and Zhou et al. reported no effect of neoadjuvant chemotherapy on HAS in a retrospective analysis.^5^, ^29^ To our knowledge, there are no reports showing the efficacy of immune checkpoint inhibitors or hyperthermic intraperitoneal chemotherapy in the literature. We introduced conventional chemotherapy for GC according to the literature^15^, ^16^, ^17^ and the result was better than reported in previous literatures. These results suggest that administering chemotherapy for GC in patients with HAS may be beneficial.

Chemotherapy is the mainstay of treatment for liver metastasis after gastrectomy and the efficacy of hepatectomy in such cases in controversial. A latest systematic review reported 1‐ and 3‐year survival rates of 69.8% and 34.8%, respectively, after hepatectomy for liver metastases of GC.^30^ In addition, in Japanese treatment guidelines, hepatectomy is weakly recommended for solitary liver metastasis without other incurable factors.^31^

In a retrospective study, Kinoshita et al. reported 1‐, 3‐, and 5‐year survival rates of 77.3%, 41.9%, and 31.1%, respectively, in a multicenter study of resection of 256 postoperative liver metastases from GC.^32^ Moreover, Oki et al. reported 3‐ and 5‐year survival rates of 51.4% and 42.3%, respectively, in 94 cases of postoperative liver metastases from GC treated with a combination of surgery, RFA, and microcoagulation therapy in a multicenter study.^33^ Unlike liver metastases from colorectal cancer, liver metastases from GC are often unresectable; therefore, the effectiveness of hepatectomy is debatable. However, these two reports suggest that hepatectomy may be beneficial in some instances. Kinoshita et al. identified three prognostic factors: the presence of serosal invasion of the primary tumor, two or fewer liver metastases, and a maximum tumor diameter of less than 5 cm. Oki et al. reported the following three prognostic factors: only one liver metastasis, up to N1 lymph node metastasis, and a maximum tumor diameter of less than 3 cm.

Although it remains unclear whether these two reports encompass HAS cases, when applying the prognostic factors from both reports to our five cases with liver metastasis, one of the five cases met the two prognostic factors reported by Kinoshita et al. and the two prognostic factors reported by Oki et al. (Table 3). Since only one patient died after recurrence, these two reports might be applicable to determine prognostic factors in instances of liver metastasis resection from GC, including HAS. Moreover, liver function was favorable in the five patients who underwent resection, and chemotherapy proved effective (Table 3). To the best of our knowledge, this is the first report addressing treatment considerations and prognosis, including hepatectomy, for recurrent liver metastases following gastrectomy for HAS. In our study, unlike in previous reports, HAS exhibited a relatively favorable prognosis. These results also suggest that aggressive hepatectomy combined with chemotherapy may improve the prognosis of recurrent liver metastases if liver function is satisfactory and the tumor is resectable. However, liver metastasis was observed in six patients (31.6%) even at pStage I after gastrectomy, implying that patients with HAS should be monitored as closely as those with advanced GC. In fact, though the difference was not statistically significant, pStage I had a worse prognosis than pStage II, III, and IV (Figure 3). The introduction of adjuvant chemotherapy for pStage I cases may lead to a lower recurrence rate and improved prognosis.

This study had some limitations. There is possibility that some AFP‐negative cases may harbor cases diagnosed as HAS by other immunostaining, because we focused on AFP‐positive HAS cases. In addition, unlike previous reports, our results are derived from a multicenter study, and our findings cannot be generalized as a strategy due to the small number of cases. Nevertheless, our report may contribute to the selection of strategies for treating liver metastases or chemotherapy in patients with HAS.

## 5 CONCLUSION

The results of our study suggest that HAS treatment according to the standard treatment guidelines for GC may result in an improved prognosis compared to previous reports. Moreover, pStage I cases also show a high recurrence rate; therefore the introduction of adjuvant chemotherapy and a follow‐up regimen similar to that used for pStage II or higher cases are desirable. Active surgical intervention for liver metastases from HAS may improve the prognosis.

## AUTHOR CONTRIBUTIONS

**Senichiro Yanagawa:** Conceptualization; methodology; formal analysis; investigation; data curation; writing – original draft; writing – review and editing; visualization. **Kazuaki Tanabe:** Conceptualization; methodology; investigation; data curation; writing – review and editing; project administration. **Mikihiro Kano:** Resources; writing – review and editing. **Ryuichi Hotta:** Resources; writing – review and editing. **Yoshihiro Saeki:** Resources; writing – review and editing. **Nobuaki Fujikuni:** Resources; data curation; writing – review and editing. **Hideki Ohdan:** Writing – review and editing; supervision.

## CONFLICT OF INTEREST STATEMENT

The authors have stated explicitly that there are no conflicts of interest in connection with this article.

## ETHICS STATEMENT

This study was conducted in accordance with the Declaration of Helsinki and approved by the Institutional Review Board of the Onomichi General Hospital Ethics Review Committee (OJH‐202254).

## Data Availability

The data analyzed during the current study are available from the corresponding author on reasonable request.

## References

[1] Zou M , Li Y , Dai Y , et al. AFP‐producing hepatoid adenocarcinoma (HAC) of peritoneum and omentum: a case report and literature review. Onco Targets Ther. 2019;12:7649‐7654. doi:10.2147/OTT.S216501 31571915 PMC6756369

[2] Søreide JA . Therapeutic approaches to gastric hepatoid adenocarcinoma: current perspectives. Ther Clin Risk Manag. 2019;15:1469‐1477. 31920320 10.2147/TCRM.S204303 PMC6934111

[3] Bosman FT , Carneiro F , Hruban RH , Theise ND . Tumours of the Stomach. WHO Classification of Tumours of the Digestive system. Vol 3. 4th ed. WHO; 2010:45‐58.

[4] Zeng XY , Yin YP , Xiao H , et al. Clinicopathological characteristics and prognosis of hepatoid adenocarcinoma of the stomach: evaluation of a pooled case series. Curr Med Sci. 2018;38:1054‐1061. doi:10.1007/s11596-018-1983-1 30536069

[5] Xia R , Zhou Y , Wang Y , Yuan J , Ma X . Hepatoid adenocarcinoma of the stomach: current perspectives and new developments. Front Oncol. 2021;11:633916. doi:10.3389/fonc.2021.633916 33912455 PMC8071951

[6] Yang X , Wang A , Li J , et al. Prognostic significance of preoperative serum tumor markers in hepatoid adenocarcinoma of stomach (HAS). BMC Cancer. 2023;23(1):53. doi:10.1186/s12885-023-10516-y 36647059 PMC9841701

[7] Li L , Yang X , Ji W , et al. Emphasis on the clinical relationship between alphas‐fetoprotein and hepatoid adenocarcinoma of the stomach: a retrospective study. BMC Gastroenterol. 2023;23(1):142. doi:10.1186/s12876-023-02773-9 37161409 PMC10170827

[8] Zhang ZR , Wu J , Li HW , Wang T . Hepatoid adenocarcinoma of the stomach: thirteen case reports and review of literature. World J Clin Cases. 2020;26(8):1164‐1171. doi:10.12998/wjcc.v8.i6.1164 PMC7103972 32258088

[9] Xie Y , Zhao Z , Li P , et al. Hepatoid adenocarcinoma of the stomach is a special and easily misdiagnosed or missed diagnosed subtype of gastric cancer with poor prognosis but curative for patients of pN0/1: the experience of a single center. Int J Clin Exp Med. 2015;8:6762‐6772. 26221214 PMC4509159

[10] Fakhruddin N , Bahmad HF , Aridi T , et al. Hepatoid adenocarcinoma of the stomach: a challenging diagnostic and therapeutic disease through a case report and review of the literature. Front Med (Lausanne). 2017;4:164. doi:10.3389/fmed.2017.00164 29034239 PMC5627014

[11] Liu JX , Wang ZK , Hong QQ , et al. Assessment of clinicopathological characteristics and development of an individualized prognostic model for patients with hepatoid adenocarcinoma of the stomach. JAMA Netw Open. 2021;4(10):e2128217. 34609494 10.1001/jamanetworkopen.2021.28217 PMC8493440

[12] Hasegawa K , Takemura N , Yamashita T , et al. Clinical practice guidelines for hepatocellular carcinoma: the Japan society of hepatology 2021 version (5th JSH‐HCC guidelines). Hepatol Res. 2023;53(5):383‐390. doi:10.1111/hepr.13892 36826411

[13] Kanda Y . Investigation of the freely available easy‐to‐use software ‘EZR’ for medical statistics. Bone Marrow Transplant. 2013;48:452‐458. doi:10.1038/bmt.2012.244 23208313 PMC3590441

[14] Liu JY , Peng CW , Yang XJ , Huang CQ , Li Y . The prognosis role of AJCC/UICC 8th edition staging system in gastric cancer, a retrospective analysis. Am J Transl Res. 2018;10(1):292‐303. 29423014 PMC5801367

[15] Bang YJ , Kim YW , Yang HK , et al. Adjuvant capecitabine and oxaliplatin for gastric cancer after D2 gastrectomy (CLASSIC): a phase 3 open‐label, randomized controlled trial. Lancet. 2012;379(9813):315‐321. 22226517 10.1016/S0140-6736(11)61873-4

[16] Sakuramoto S , Sasako M , Yamaguchi T , et al. Adjuvant chemotherapy for gastric cancer with S‐1, an oral fluoropyrimidine. N Engl Med. 2007;357(18):1810‐1820. 10.1056/NEJMoa072252 17978289

[17] Yoshida K , Kodera Y , Kochi M , et al. Addition of docetaxel to oral fluoropyrimidine improves efficacy in patients with stage III gastric cancer: interim analysis of JACCRO GC‐07, a randomized controlled trial. J Clin Oncol. 2019;37(15):1296‐1304. 30925125 10.1200/JCO.18.01138 PMC6524985

[18] Becker K , Mueller JD , Schulmacher C , et al. Histomorphology and grading of regression in gastric carcinoma treated with neoadjuvant chemotherapy. Cancer. 2003;98:1521‐1530. doi:10.1002/cncr.11660 14508841

[19] Thies S , Langer R . Tumor regression grading of gastrointestinal carcinomas after neoadjuvant treatment. Front Oncol. 2013;3:262. doi:10.3389/fonc.2013.00262 24109590 PMC3791673

[20] Ishikura H , Fukasawa Y , Ogasawara K , Natori T , Tsukada Y , Aizawa M . An AFP‐producing gastric carcinoma with features of hepatic differentiation: a case report. Cancer. 1985;56:840‐848. doi:10.1002/1097-0142(19850815)56:4<840::aid-cncr2820560423>3.0.co;2-e 2410093

[21] Zhang JF , Shi SS , Shao YF , Zhang HZ . Clinicopathological and prognostic features of hepatoid adenocarcinoma of the stomach. Chin Med J (Engl). 2011;124:1470‐1476. 21740800

[22] Liu X , Sheng W , Wang Y . An analysis of clinicopathological features and prognosis by comparing hepatoid adenocarcinoma of the stomach with AFP‐producing gastric cancer. J Surg Oncol. 2012;106:299‐303. doi:10.1002/jso.23073 22389029

[23] Yang J , Wang R , Zhang W , Zhuang W , Wang M , Tang C . Clinicopathological and prognostic characteristics of hepatoid adenocarcinoma of the stomach. Gastroenterol Res Pract. 2014;2014:140587. doi:10.1155/2014/140587 24669215 PMC3942340

[24] Lin CY , Yeh HC , Hsu CM , Lin WR , Chiu CT . Clinicopathological features of gastric hepatoid adenocarcinoma. Biom J. 2015;38:65‐69. doi:10.4103/2319-4170.126860 25163499

[25] Xiao C , Wu F , Jiang H , et al. Hepatoid adenocarcinoma of the stomach: nine case reports and treatment outcomes. Oncol Lett. 2015;10:1605‐1609. doi:10.3892/ol.2015.3430 26622718 PMC4533739

[26] Wang Y , Sun L , Li Z , et al. Hepatoid adenocarcinoma of the stomach: a unique subgroup with distinct clinicopathological and molecular features. Gastric Cancer. 2019;22:1183‐1192. doi:10.1007/s10120-019-00965-5 30989433 PMC6811386

[27] Zhou K , Wang A , Ao S , et al. The prognosis of hepatoid adenocarcinoma of the stomach: a propensity score‐based analysis. BMC Cancer. 2020;20:671. doi:10.1186/s12885-020-07031-9 32680468 PMC7368673

[28] Simmet V , Noblecourt M , Lizée T , et al. Chemotherapy of metastatic hepatoid adenocarcinoma: literature review and two case reports with cisplatin etoposide. Oncol Lett. 2018;15:48‐54. doi:10.3892/ol.2017.7263 29387209 PMC5769300

[29] Zhou K , Wang A , Wei J , et al. The value of perioperative chemotherapy for patients with hepatoid adenocarcinoma of the stomach undergoing radical gastrectomy. Front Oncol. 2022;10(11):789104. doi:10.3389/fonc.2021.789104 PMC8784750 35083146

[30] Monroy DC , Ibanez‐Pinilla M , Sabogal JC , et al. Survival outcomes of hepatectomy in gastric cancer liver metastasis: a systematic review and meta‐analysis. J Clin Med. 2023;12:704. 36675632 10.3390/jcm12020704 PMC9861719

[31] Japanese Gastric Cancer Association . Japanese gastric cancer treatment guidelines 2021 (6th edition). Gastric Cancer. 2023;26:1‐25. 36342574 10.1007/s10120-022-01331-8 PMC9813208

[32] Kinoshita T , Kinoshita T , Saiura A , Esaki M , Sakamoto H , Yamanaka T . Multicentre analysis of long‐term outcome after surgical resection for gastric cancer liver metastases. Br J Surg. 2015;102:102‐107. doi:10.1002/bjs.9684 25389030

[33] Oki E , Tokunaga S , Emi Y , et al. Surgical treatment of liver metastasis of gastric cancer: a retrospective multicenter cohort study (KSCC1302). Gastric Cancer. 2016;19:968‐976. doi:10.1007/s10120-015-0530-z 26260876

